# Pattern of Morphological Variability in Unrepaired Unilateral Clefts With and Without Cleft Palate May Suggest Intrinsic Growth Deficiency

**DOI:** 10.3389/fcell.2020.587859

**Published:** 2020-12-11

**Authors:** Benny S. Latief, Mette A. R. Kuijpers, Adam Stebel, Anne Marie Kuijpers-Jagtman, Piotr S. Fudalej

**Affiliations:** ^1^Department of Oral and Maxillofacial Surgery, Faculty of Dentistry, Universitas Indonesia, Jakarta, Indonesia; ^2^Department of Dentistry – Orthodontics and Craniofacial Biology, Radboud University Medical Center, Radboud Institute for Health Sciences, Nijmegen, Netherlands; ^3^Department of Maxillofacial Surgery, F. D. Roosevelt University Hospital, Banská Bystrica, Slovakia; ^4^Department of Stomatology and Maxillofacial Surgery, Faculty of Medicine, Comenius University, Bratislava, Slovakia; ^5^Faculty of Dentistry, Universitas Indonesia, Jakarta, Indonesia; ^6^Department of Orthodontics, University Medical Center Groningen, Groningen, Netherlands; ^7^Department of Orthodontics and Dentofacial Orthopedics, University of Bern, Bern, Switzerland; ^8^Department of Orthodontics, Jagiellonian University, Kraków, Poland; ^9^Department of Orthodontics, Institute of Dentistry and Oral Sciences, Palacký University Olomouc, Olomouc, Czechia

**Keywords:** unilateral cleft lip and palate, unilateral cleft lip, unrepaired clefts, geometric morphometrics, maxillary growth, facial morphology intrinsic growth deficiency in clefts

## Abstract

In individuals with cleft lip and palate (CLP) an iatrogenic effect of operations on subsequent maxillary growth is well-known. Much less is known about the association between occurrence of CLP and intrinsic growth deficiency of the maxillofacial complex. The aim of this study was to compare morphological variability in subjects with unilateral cleft lip and alveolus/palate and unaffected controls using geometric morphometric methods. The research hypothesis was that if subjects with *unrepaired* unilateral CLP have intrinsic growth deficiency, the pattern of their craniofacial growth variation may differ from that in unaffected individuals. Lateral cephalograms were available of three groups of the same ethnic background (Proto-Malayid): (a) non-syndromic *unrepaired* unilateral complete cleft lip, alveolus, and palate (UCLP), *N* = 66, mean age 24.5 years (b) non-syndromic *unrepaired* unilateral complete cleft lip and alveolus (UCLA), *N* = 177, mean age 23.7 years, and (c) NORM (*N* = 50), mean age 21.2 years without a cleft. Using geometric morphometrics shape variability in groups and shape differences between groups was analyzed. Principal component analysis (PCA) was used to examine shape variability, while differences between groups and sexes were evaluated with canonical variate analysis. Sexual dimorphism was evaluated with discriminant function analysis (DA). Results showed that in comparison to NORM subjects, shape variability in UCLA and UCLP is more pronounced in the antero-posterior than in vertical direction. Pairwise comparisons of the mean shape configurations (NORM vs. UCLA, NORM vs. UCLP, and UCLA vs. UCLP) revealed significant differences between cleft and non-cleft subjects. The first canonical variate (CV1, 68.2% of variance) demonstrated that differences were associated with maxillary shape and/or position and incisor inclination, while in females, the CV1 (69.2% of variance) showed a combination of differences of “maxillary shape and/or position and incisor inclination” and inclination of the cranial base. Shape variability demonstrated considerable differences in subjects with UCLA, UCLP, and NORM. Moreover, in subjects with a cleft, within-sample variability was more pronounced in the antero-posterior direction, while in non-cleft subjects, within-sample variability was more pronounced in the vertical direction. These findings may suggest that subjects with unilateral clefts have intrinsic growth impairment affecting subsequent facial development.

## Introduction

Cleft lip with or without cleft palate shows a large phenotypic variation ranging from complete open clefts of the lip, alveolus and palate to microforms and subclinical phenotypes like submucous cleft lip or palate. All of them have their own specific phenotype and the related problems are different for each type of cleft. Surgical rehabilitation of patients with cleft lip and palate aims at restoration of the anatomy, function, and aesthetics of the face, but is associated with growth disturbance of the midface. Identification of factor(s) leading to maxillofacial growth disturbance in individuals with cleft lip and palate (CLP) is critical for improvement of treatment results. To date, numerous animal experiments have shown that scar tissue that develops after the palatal surface has been denuded to close the cleft and the palate, is a strong inhibitor of maxillary growth and the adverse growth effect persists into adulthood (Li et al., 2015).

Much less is known about the association between occurrence of CLP and intrinsic growth deficiency of the maxillofacial complex. The theoretical ground for it is that the processes causing lack of fusion of maxillary/nasal prominences during embryonic life could also lead to impaired growth. One way to assess this would be to compare craniofacial morphology in subjects with unoperated CLP with their non-cleft counterparts. However, for obvious reasons it is difficult to collect a large sample of untreated CLP individuals. Several investigations on untreated clefts produced conflicting results and demonstrated that the maxilla could be smaller (Capelozza et al., 1993; Liao and Mars, 2005), comparable (Shetye and Evans, 2006; Diah et al., 2007), or larger (Ortiz-Monasterio et al., 1974) than in non-cleft controls. Data for unoperated bilateral clefts is even more scarce and comprise mostly case reports (Will, 2000). These inconsistent findings may be due to the error of the method, different ages of evaluation, or the use of samples with mixed cleft types.

An alternative approach is a comparison of variability of craniofacial morphology in subjects with unoperated CLP with non-cleft individuals using geometric morphometry, a statistical method to analyze shape (Halazonetis, 2004; Zelditch et al., 2012). This method has been used in a few studies comparing two- or three-dimensionally craniofacial morphology of patients operated for a cleft, but comparisons between individuals with unoperated clefts and non-cleft controls are scarce (Toro-Ibacache et al., 2014; Liberton et al., 2020). Our recent study (Latif et al., 2020) demonstrated differences in facial variation between subjects with unrepaired bilateral clefts and non-cleft controls. Variability was mainly present in the vertical direction in non-cleft subjects, while in bilateral CLP subjects the anteroposterior component of variation was marked. We suggested that this difference might point to intrinsic growth impairment in bilateral CLP.

The aim of this study was to compare facial morphological variability in subjects with unilateral cleft lip and alveolus/palate and unaffected controls using geometric morphometric methods. The research hypothesis (H_R_) tested in this study was that the patterns of craniofacial shape variations in subjects with unilateral cleft lip and alveolus/palate and unaffected controls is different.

## Subjects and Methods

### Sample

The study sample consisted of three groups from the same ethnic background (Proto-Malayid): (**a**) group comprising 66 subjects (37 males, 29 females, mean age 24.5 years, SD 10.5, range 14–61 years) with a non-syndromic *unrepaired* unilateral complete cleft lip, alveolus, and palate (UCLP group), (**b**) group comprising 177 subjects (104 males, 73 females, mean age 23.7 years, SD 10.9, range 14–72 years) with a non-syndromic *unrepaired* unilateral complete cleft lip and alveolus (UCLA group), and (**c**) group comprising 50 subjects (25 males, 25 females, mean age 21.2 years, SD 3.2, range 15–31 years)—NORM group.

The cleft sample was collected between 1986 and 1997 during nine charity missions in the province of East Nusa Tenggara, Indonesia, as part of a larger study as described earlier (Latief et al., 2010) in cooperation between the University of Brawijaya, Faculty of Medicine (Malang, Indonesia), Universitas Indonesia, Faculty of Dentistry (Jakarta, Indonesia), University Medical Centre Leiden, Department of Oral and Maxillofacial Surgery (Leiden, Netherlands) and the Radboud University Medical Centre (Nijmegen, Netherlands). The inclusion criteria were Proto-Malayid origin, complete unrepaired non-syndromic UCLA or UCLP, non-syndromic cleft as ascertained from medical records and clinical examination; the exclusion criteria were additional submucosal cleft and/or Simonart’s band, earlier surgical repair or orthodontic treatment, lack of adequate contacts between opposing molars, age younger than 14 years.

The NORM group was collected in 1997 and consisted of 50 healthy volunteers from the city of Kupang (capital of the province East Nusa Tenggara). The inclusion criteria were Proto-Malayid origin, no cleft or other craniofacial anomaly, no clefts in the family history, normal occlusal relationship (Angle Class I); The exclusion criteria were earlier orthodontic or surgical treatment in the maxillofacial region, and age younger than 14 years. All study participants (clefts and controls) signed the informed consent.

The Bioethics Committee of the University of Indonesia approved this investigation in 2015 (Ref #: *1/EthEx/FKGUI/II/2015*).

### Methods

Lateral cephalograms taken prior to surgical closure of the cleft were available for further analysis. The radiographs were taken in a mobile custom-built radiographic setup including a cephalostat with a focus-film distance of 1.70 m. The film cassette was 24 × 30 cm with a high-speed intensifying screen to shorten the exposure time. Considering field conditions during imaging of cleft and non-cleft subjects, it is likely that the radiographic magnification factor could not have been kept constant. Therefore, only facial shape (i.e., without information about the size) was evaluated in this study. The cephalograms were scanned at 300 dpi resolution.

**FIGURE 1:**
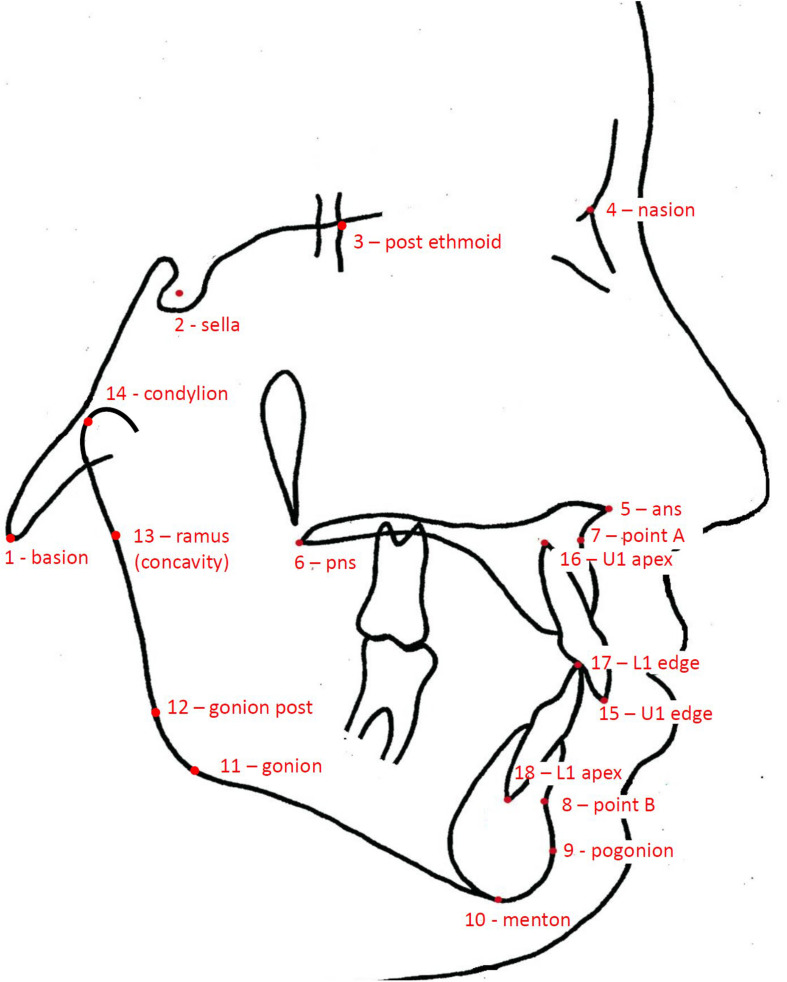
Drawing of a cephalogram illustrating the anatomical landmarks used in this study.

Facial morphology was evaluated on lateral radiographs of the head (cephalograms). Because the contour of the soft tissues was blurred and difficult to identify, the assessment of the facial morphology was limited to bony structures. The geometry of the cranial base, the maxillary complex, the mandible, and the anterior dentition was captured using 18 anatomical landmarks (Figure 1). We used the same landmarks as in the study by Latif et al. (2020), which seemed sufficient to represent key anatomical structures of the craniofacial skeleton.

The landmarks were digitized by one investigator (PF) on the scan of each radiograph with the tpsDig2 program, version 2.18^1^. Two-dimensional landmark coordinates were extracted and exported in TPS format to be used for the geometric morphometric analyses with the MorphoJ software package, version 1.06d (Klingenberg, 2011). The sample was checked for outliers—none was detected.

To assess the intra-observer reliability 28 images were randomly selected and re-digitized by the same investigator (PF) after a minimum of 1 month. Random error was expressed as the Procrustes distance between the redigitizations in shape space in comparison with the total shape variance.

### Statistical Analysis

Two areas were analyzed in this investigation: shape variability in the groups and shape differences between the groups. First, principal component analysis (PCA) was used to examine shape variability. The effect of group, age, and sex on the shape was evaluated with multivariate regression analysis, where Procrustes shape coordinates were dependent variables and the group, age, and sex were covariates. Differences between the UCLP, UCLA, and NORM groups, i.e., the Procrustes distances between group means, for males and females were assessed using canonical variate analysis (CVA), which is a method used to find the shape features that best distinguish among multiple groups of subjects (Zelditch et al., 2012). Sexual dimorphism in the UCLA, UCLP, and NORM groups was evaluated with discriminant function analysis (DA; equivalent to CVA but used for comparisons between two groups only). Analogous as in CVA, also in DA the Procrustes distances between males and females were established.

All analyses and statistical tests were performed in MorphoJ and PAST v.3 software (Øyvind Hammer, University of Oslo, Norway). Permutation tests (100,000 permutation runs) with a significance level of 0.05 were used to establish intergroup differences in facial shapes. The visualization of facial shape changes was carried out in MorphoJ and Viewbox 4.1 (dHAL Software, Kifissia, Greece).

## Results

### Method Error

The measurement error was relatively small in the redigitized subsample—it was equal to 7.29% of the total variation.

### Within-Sample Shape Variability

**FIGURE 2:**
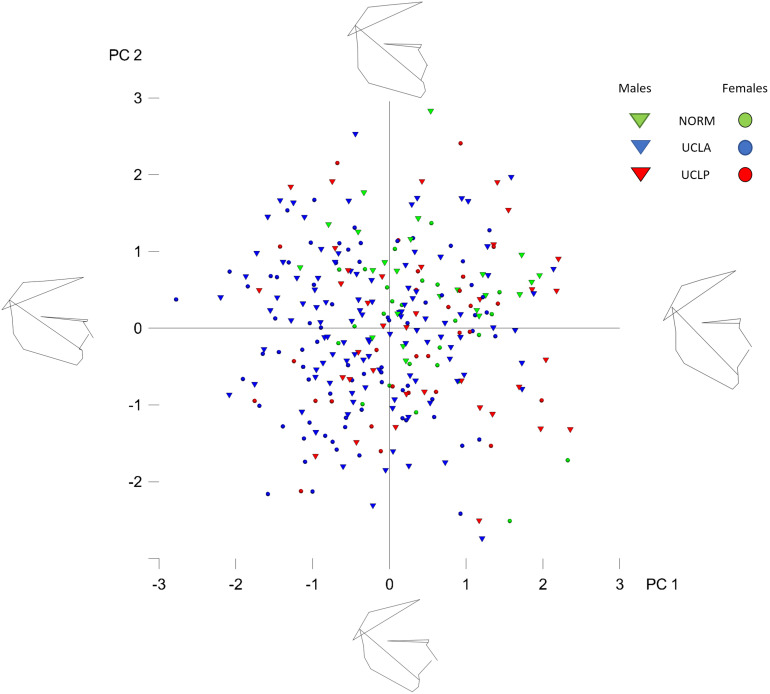
Principal component analysis of shape variables in the whole sample (subjects with and without cleft pooled together). The figure presents the variation in the whole sample along PC axes 1 and 2 (PC1 and PC2). Each “point” represents a single subject. The numbers (1, 2, 3, -1, -2, -3) on the axes denote standard deviation (SD).

**FIGURE 3:**
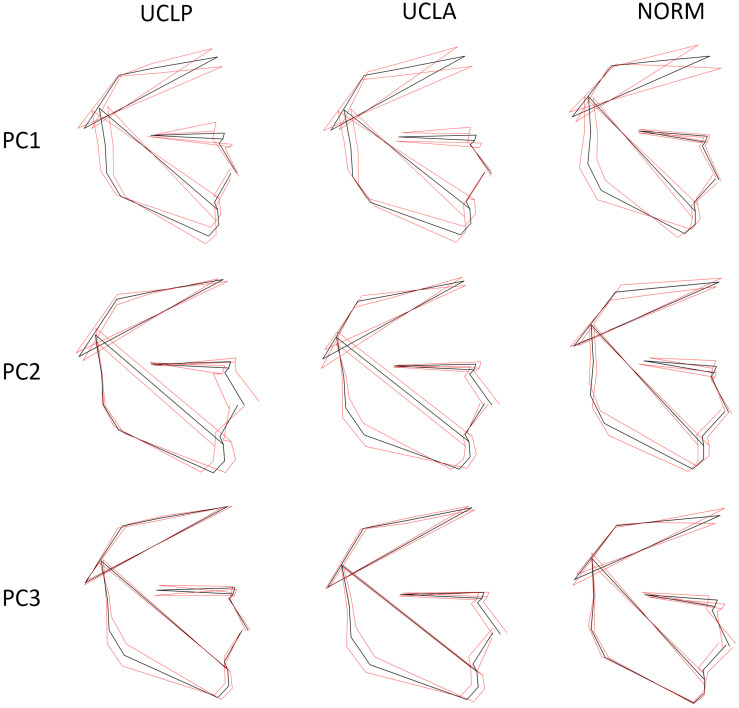
Variation in subjects with unilateral cleft lip and palate (UCLP), unilateral cleft lip and alveolus (UCLA), and in unaffected subjects (NORM) along the first three principal components (PC1–PC3). The black shape is the mean shape, the red shapes correspond with the shape at -3 SD and +3 SD for each axis (PC1–PC3) and each group (UCLP, UCLA, NORM).

The plot depicting overall shape variability in the sample (Figure 2) implies shape differences between groups. The pattern of scatter of individual shapes with a concentration of NORM shapes in the upper right region of the plot (i.e., PC1 and PC2 are > 0) and few NORM shapes in the lower left region of the plot (i.e., PC1 and PC2 are < 0) suggests that non-cleft subjects differ from subjects with a cleft. The principal components 1 through 5, each accounting for at least 5% of variance, explained in total 60.3% of variance among individuals (Table 1 and Supplementary Table 1 presents variance explained by all principal components). The first major axis of shape variation (PC1, 21.3% of variance) demonstrates shape patterns in the vertical direction, while PC2 (14.1% of variance) depicts mainly anteroposterior shape patterns, particularly of the size and/or position of the mandible relative to the cranial base and maxillary complex. Figure 3 shows shape variability separately in UCLP, UCLA, and NORM groups. In comparison to non-cleft subjects, shape variability in individuals with unilateral clefts is more pronounced in the antero-posterior than in vertical direction. Supplementary Table 2 demonstrates variability for each landmark in x and y directions.

**TABLE 1:** Proportion of variance in a sample comprising subjects with clefts (UCLP and UCLA) (*N* = 243) and without clefts (*N* = 50) described by principal components (PCs), explaining at least 5% of variance each, in shape space.

Principal component (PC)	% Variance	Cumulative %
PC1	21.3%	21.3%
PC2	14.1%	35.4%
PC3	11.8%	47.2%
PC4	8.1%	55.3%
PC5	5%	60.3%
Remaining PCs	*39.7%*	*100%*

### Age-Shape Correlation

Regression analysis (Table 2) with group, sex, and age as covariates and PC1–PC5 as dependent variables showed a limited effect of the age on the facial shape variability—the age “weighted” considerably less than group and sex. This indicates that a subject’s age was not a predictor of the facial shape, either in males or in females, or with or without a cleft.

**TABLE 2:** Multivariate regression analysis with group, sex, and age as covariates (independent variables) and principal components 1–5 (PC1–PC5) as dependent variables.

		Coeff.	Std. Err.	*p*	*R*^2^
pc1	Sex	−6.51	4.07	0.110	0.009
	Age	−0.08	0.20	0.706	0.000
	Group	0.30	3.24	0.925	0.000
pc2	Sex	−7.94	3.20	0.014	0.023
	Age	0.50	0.16	0.002	0.031
	Group	−5.64	2.55	0.028	0.011
pc3	Sex	−9.93	2.91	0.001	0.043
	Age	0.52	0.15	0.000	0.046
	Group	−1.80	2.32	0.439	0.000
pc4	Sex	−6.25	2.46	0.012	0.023
	Age	0.01	0.12	0.910	0.001
	Group	4.35	1.96	0.027	0.018
pc5	Sex	−4.16	1.79	0.021	0.014
	Age	−0.38	0.09	0.000	0.034
	Group	9.31	1.43	0.000	0.111

*Wilks λ = 0.6596; F: 8.508; df1: 15; df2: 784.4; p-value (the whole model) < 0.001.*

*Tests on independent variables:*

* (1) Sex: Wilks λ = 0.894; p < 0.001.*

* (2) Age: Wilks λ = 0.862; p < 0.001.*

* (3) Group: Wilks λ = 0.832; p < 0.001.*

*Tests on dependent variables:*

* (1) pc1: R^2^ = 0.009, p = 0.451.*

* (2) pc2: R^2^ = 0.066, p < 0.001.*

* (3) pc3: R^2^ = 0.084, p < 0.001.*

* (4) pc4: R^2^ = 0.04, p = 0.008.*

* (5) pc5: R^2^ = 0.1749, p < 0.001.*

### Sexual Dimorphism

**FIGURE 4:**
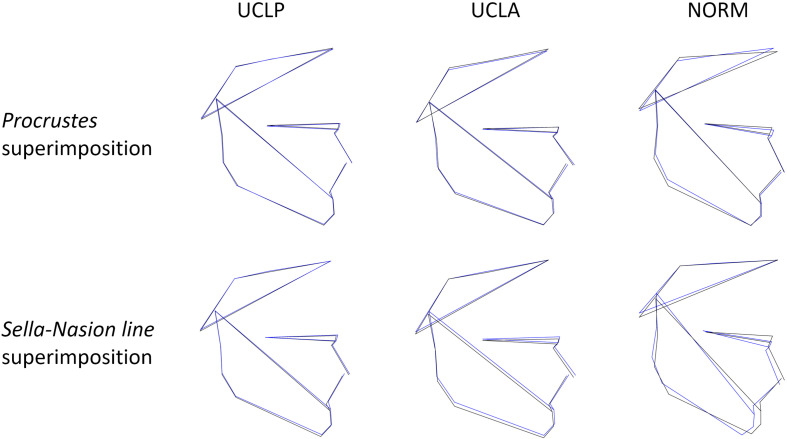
Visualization of sexual dimorphism in subjects with unilateral cleft lip and palate (UCLP), unilateral cleft lip and alveolus (UCLA), and in unaffected subjects (NORM) based on discriminant function analysis. Black color—males, blue—females.

Sexual dimorphism was detected in UCLA and NORM groups but not in UCLP group (Table 3). The Procrustes distance between mean shapes for males and females was largest for non-cleft subjects (NORM) and smallest for subjects of the UCLP group. The non-cleft males had a shorter anterior facial height and flatter mandibular plane than females (Figure 4), while the males and females with UCLA had subtle differences in vertical proportions.

**TABLE 3:** Sexual dimorphism in groups assessed with discriminant function analysis (DA).

	Groups		
	
	UCLP	UCLA	NORM
Procrustes distance	0.01405	0.02072	0.04035
95%CI	0.01212–0.02713	0.00706–0.01587	0.01217–0.02759
*p*-value	0.896	<0.001	<0.001

*P-values for permutation tests (100,000 permutation runs).*

### Differences Between UCLP, UCLA, and NORM Groups

**FIGURE 5:**
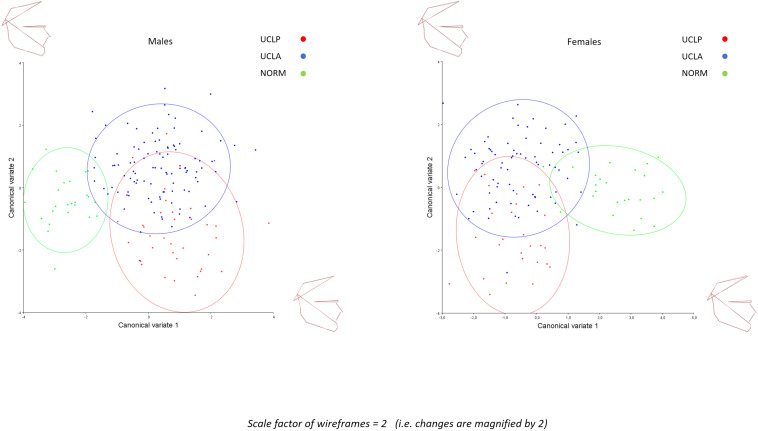
Canonical variate analysis of males and females with unilateral cleft lip and palate (UCLP), with unilateral cleft lip and alveolus (UCLA), and in unaffected individuals (NORM).

The inter-group differences between the mean shape configurations in each group were analyzed with CVA (Table 4). Pairwise comparisons (NORM vs. UCLA, NORM vs. UCLP, and UCLA vs. UCLP) in males and females revealed statistically significant differences between subjects with and without a cleft. In males, the first canonical variate (CV1, 68.2% of variance) demonstrated that differences were associated with maxillary shape and/or position and incisor inclination, while in females, the CV1 (69.2% of variance) showed a combination of differences of “maxillary shape and/or position and incisor inclination” and inclination of the cranial base (Figure 5).

**TABLE 4:** Pairwise differences between groups in facial shape configurations assessed with canonical variate analysis (CVA).

		**UCLA**	**UCLP**
Both sexes	NORM	0.0353 (*p* < 0.001)	0.0311 (*p* < 0.001)
	UCLA		0.0286 (*p* < 0.001)
Males	NORM	0.0373 (*p* < 0.001)	0.0386 (*p* < 0.001)
	UCLA		0.0319 (*p* < 0.001)
Females	NORM	0.041 (*p* < 0.001)	0.0371 (*p* < 0.001)
	UCLA		0.0313 (*p* < 0.001)

*P-values from permutation tests (100,000 permutation runs) in brackets.*

## Discussion

In the current study we used geometric morphometrics (GM) to evaluate craniofacial shape. This novel method has been commonly used in anatomy and anthropology. We are aware that the vast majority of studies evaluating craniofacial shape in cleft and non-cleft population has used conventional cephalometrics as a shape descriptor. However, cephalometric analysis has inherent problems regarding its applicability as shape measure; it provides only a partial and localized description of shape and is confounded by our biases regarding the reference structures. As a result (a) the measurements are often conflicting, (b) many measurements are needed for a comprehensive shape description, (c) it is not trivial to compare the craniofacial pattern between different individuals, and (d) classification of shape is based on a limited subset of all possible measurements and might therefore be biased by that particular selection. In comparison to conventional cephalometrics, GM allows for (a) comprehensive description of the overall craniofacial shape with measurements, which are not conflicting because they are unrelated statistically; (b) easy assessment of the degree of variation from the mean; (c) it is relative insensitive to errors in landmark identification (Halazonetis, 2004). These advantages make GM an interesting alternative in studies of the craniofacial shape (Wellens et al., 2013; Urbanova et al., 2016; Jaklová et al., 2020; Segna et al., 2020). Unfortunately, the “non-intuitive” mathematical methods of GM can present a challenge in interpretation of results.

Studies exploring the craniofacial shape in humans without congenital malformations (Rosas et al., 2008; Wellens and Kuijpers-Jagtman, 2016; Katsadouris and Halazonetis, 2017; Paoloni et al., 2017) demonstrated that the largest variability of the shape concerned the vertical direction (i.e., *dolichocephalic* vs. *brachycephalic* shape) irrespective of the ethnic background or population origin. Although contemporary populations from Europe are more *dolichocephalic* than those coming from Asia (African populations are relatively most *brachycephalic)*, vertical variability (i.e., different degree of *dolichocephalic*/*brachycephalic* facial pattern) prevails over variability in antero-posterior direction (Rosas et al., 2008). Thus, our research hypothesis was that if subjects with unilateral cleft lip and palate do have intrinsic growth deficiency, the pattern of their craniofacial growth variation may differ from the pattern of variation in unaffected individuals.

In our previous study on individuals with bilateral orofacial clefts, we found evidence that subjects with unrepaired *bilateral* CLA and *bilateral* CLP did demonstrate a disparate pattern of craniofacial variation. Unaffected subjects and individuals with a bilateral cleft differed mostly in respect to the position of the (pre)maxilla, which was more protruded in the BCLA and BCLP groups than in controls (Latif et al., 2020). The aim of the current study was to test whether the difference in craniofacial shape variation between subjects with unrepaired unilateral orofacial clefts and their non-affected counterparts is comparable to the disparity between subjects with bilateral orofacial clefts and unaffected ones.

In the present study we found that subjects with a unilateral cleft had a considerably different pattern of craniofacial variation. In UCLA and UCLP groups PC1 through PC3 (cumulative variance: 47.2%) depicted a variation in both vertical and anteroposterior direction. Both directions of variation are easily discernible in Figure 3, with a possible exception of PC3 in the UCLP group, where anteroposterior variation of the maxilla was limited. In the NORM group, in contrast, variation in anteroposterior direction along the axes for PC1 and PC2 was limited, particularly for the maxilla (Figure 3). Furthermore, comparison of patterns of variation between subjects with different cleft types (UCLA and UCLP groups) demonstrates a lack of significant differences between the groups. PC1 shows an almost identical pattern of variation in the UCLA and UCLP groups, while PC2 indicates more anteroposterior variation in UCLP than in the UCLA group. The similarity of patterns of variation for subjects with a different type of the cleft on one side and the difference in patterns of variation between subjects with and without a cleft on the other side are visualized by clear separation of groups with and without a cleft along Canonical Variate 1 axis (Figure 5). Therefore, our findings imply that the orofacial cleft may be associated with intrinsic deficiency of growth of the structures affected by the cleft.

In exploratory studies such as the present one it is impossible to identify mechanisms responsible for growth deficiency in the cleft anomaly. Instead they serve as a tool to corroborate that such growth deficiency is possible. It is particularly important in subjects with unilateral cleft lip and alveolus/palate because there is an ongoing controversy encompassing the problem of the impact of intrinsic growth deficiency vs. surgical iatrogenesis on subsequent craniofacial development. As discussed in our previous paper (Latif et al., 2020) “In theory, if facial growth deficiency is exclusively the result of treatment of the cleft deformity, one could expect that the pattern of variations of the shape of the face in untreated clefts is comparable with the pattern of variations in subjects without an orofacial cleft. If the opposite is true, the pattern of facial shape variations in untreated and treated clefts should be different.” Publications to date demonstrated somewhat contradictory results—Lambrecht et al. (2000) reviewed studies assessing craniofacial morphology in unrepaired unilateral cleft lip and palate in comparison with non-cleft individuals and found that the maxillary complex in the cleft anomaly can be retruded (one study found retrusion), normal (three studies), or protruded (six studies). Later publications (Shetye and Evans, 2006; Chen et al., 2012) implied that in unrepaired unilateral cleft lip and palate although the length of the maxilla appears to be somewhat shorter in many patients, sagittal growth does not seem to be significantly disturbed. Our findings seem to disagree with the results of these studies assessing craniofacial morphology as we found evidence for an intrinsic growth deficiency within the craniofacial complex, while most clinical studies revealed little maxillofacial growth disturbance in adults with unrepaired cleft lip and palate. There may be several explanations for this discrepancy.

First, the investigations mentioned above had rather small sample sizes which increases uncertainty for *generalizability* of findings. Second, the spatial position of the maxilla is typically assessed by measuring the angle between the point A and cranial base (or Frankfurt horizontal plane). The larger the angle, the more protruded the maxilla. However, in unrepaired cleft lip and palate when there is discontinuity of the orbicularis muscle and a wide cleft, the anterior part of the larger segment (on which point A is located) can be rotated forward. The repair of the cleft can result in maxillary remodeling and posterior dislocation of point A. In other words, location of point A in the unrepaired cleft and non-cleft maxilla is not completely homologous. Finally, conventional cephalometric analysis as used in earlier studies has significant limitations such as the use of many inter-related landmarks and angles to characterize morphology of the anatomical structure (Moyers and Bookstein, 1979; Bookstein, 2016). In effect the change of a given angle (e.g., sella-nasion-point A) cannot be precisely ascribed to the change of a structure (e.g., position of point A on the surface of maxilla) because the change of reference line (e.g., sella-nasion, dependent on position of both sella and nasion landmarks) might well be responsible for it (Halazonetis, 2004). Therefore the use of geometric morphometrics to analyze shape is a better choice.

The pattern of shape variation in subjects with unrepaired **unilateral** clefts is **similar** to that found in **bilateral** clefts (Latif et al., 2020). The variation along the PC1 axis in UCLA, UCLP, and pooled bilateral cleft lip and alveolus (BCLA)—bilateral cleft lip, alveolus, and palate (BCLP) groups is a combination of variation in vertical and anteroposterior direction. Both components (directions) are easily recognizable on the plots. A subtle difference is that the anteroposterior component in the BCLA-BCLP group is more pronounced than in UCLA and UCLP groups. This may reflect a more variable position of the pre-maxilla in subjects with bilateral clefts but could also be due to a significantly smaller sample size in the BCLA-BCLP group. The variation along the PC2 axis is also a combination of variation in the vertical and anteroposterior direction. However, in the BCLA-BCLP group the vertical component is stronger than along PC1 axis; in UCLA and UCLP groups the vertical component is weaker than the anteroposterior component, especially in subjects with UCLP. A comparison with the control group (in both studies the same group of non-cleft subjects was used) demonstrates that cleft vs. non-cleft differences are considerably larger than discrepancies between groups with different cleft types. To understand the effect of treatment and intrinsic growth deficiency it would be interesting to be able to compare a treated group with the same ethnicity and a control group.

The strengths of this investigation are a large sample size, the use of controls derived from the same ethnic group, and the use of geometric morphometrics for characterization of craniofacial variability. Nevertheless, this study has several limitations mentioned previously (Latif et al., 2020). In summary, some subjects were relatively young (i.e., 14–18 years of age) when the cephalogram was collected. The age of subjects should be viewed in the context of achievement of relative stability of craniofacial structures. Indonesian populations, as other from equatorial regions, start and finish their maturation phase earlier.

Nevertheless, the literature on unoperated cleft uses the age of 13 years to pinpoint the start of the adulthood stage (Latief et al., 2010). Certainly, the aging affects facial soft tissues; however, its effect on the skeleton is limited, when assessed with geometric morphometrics, provided a subject has adequate occlusal contacts between opposing teeth (otherwise a loss of opposing teeth can result in loss of occlusal support and decrease of facial height). In our study we excluded subjects who had missing occlusal contacts between opposing teeth. We used 2D cephalograms, although 3D imaging is currently the method of choice, but this is not a feasible option in a low-income country and an isolated population. The soft tissue profile was difficult to identify so we were restricted to analysis of the hard tissues only. In future research the presently available handheld 3D cameras may facilitate soft tissue analysis of patients and controls in remote areas. Finally, the lack of information on magnification of facial structures on cephalograms prevented us from analyzing the size and allometry in subjects with and without a cleft.

## Conclusion

Shape variability demonstrates considerable differences in subjects with unrepaired complete unilateral cleft lip and alveolus (UCLA) and complete unilateral cleft lip, alveolus and palate (UCLP) and non-cleft subjects (NORM). Moreover, in subjects with a cleft, within-sample variability was more pronounced in the antero-posterior direction, while in non-cleft subjects, within-sample variability was more pronounced in the vertical direction. These findings may suggest that subjects with unilateral clefts have intrinsic growth impairment affecting subsequent facial development.

## Data Availability Statement

The raw data supporting the conclusions of this article will be made available by the authors, without undue reservation, to any qualified researcher.

## Ethics Statement

The studies involving human participants were reviewed and approved by the Bioethics Committee of the University of Indonesia (Ref#: 1/EthEx/FKGUI/II/2015). Written informed consent for participation was not required for this study in accordance with the national legislation and the institutional requirements.

## Author Contributions

PF, MK, and AK-J conceived and designed the study. BL and AK-J collected the data. PF digitized the radiographs. PF and MK performed analyses. BL, MK, AS, AK-J, and PF contributed to the data interpretation. BL, AS, and PF wrote the draft manuscript. All authors critically revised, finalized, and approved the manuscript.

## Conflict of Interest

The authors declare that the research was conducted in the absence of any commercial or financial relationships that could be construed as a potential conflict of interest.

## Footnotes

1 http://life.bio.sunysb.edu/morph/
